# Comparative Evaluation of Antimicrobial Efficacy of Various Antibiotic Pastes and Calcium Hydroxide Using Chitosan as a Carrier Against Enterococcus faecalis: An In Vitro Study

**DOI:** 10.7759/cureus.43541

**Published:** 2023-08-15

**Authors:** Srinidhi S R, Sania Singh, Ajit Hindlekar, Niranjan Desai, Nishant Vyavahare

**Affiliations:** 1 Conservative Dentistry and Endodontics, Dr. D. Y. Patil Dental College & Hospital, Pune, IND; 2 Conservative Dentistry and Endodontics, Sinhgad Dental College & Hospital, Pune, IND

**Keywords:** intracanal medicament, enterococcus faecalis (e. faecalis), calcium hydroxide, chitosan, bacterial leakage

## Abstract

Introduction: The use of intracanal medicaments between appointments can serve as an important aid in the sterilization of the root canal system. Calcium hydroxide is commonly used, but it is not completely effective against *Enterococcus faecalis*. A triple antibiotic paste (TAP) is used but has the problem of tooth discoloration. Double antibiotic paste (DAP) or modified TAP (MTAP) has been suggested to solve this. Chitosan has been used as a vehicle in pharmacology, and it has inherent antibacterial properties too.

Objective: This study compares the efficacy of DAP and MTAP using chitosan as a vehicle with calcium hydroxide.

Materials and methods: Sixty single-rooted teeth were taken and decoronated with a length of 13 mm. Biomechanical preparation (BMP) was done with #3 Gates-Glidden (GG) drills, 17% ethylenediaminetetraacetic acid (EDTA), and 5.25% sodium hypochlorite (NaOCl) used for irrigation. These were kept in microcentrifuge tubes with 1 ml brain heart infusion (BHI) broth. Contamination was done with *E. faecalis* strain for 21 days. DAP and MTAP pastes were prepared and added to the chitosan solution. Groups were divided into 10 each, each medicament with saline or chitosan as the vehicle. The microbial load was measured at the end of two and seven days.

Results: The level of significance was kept at p = 0.05. Statistical analysis was done with the Kruskal-Wallis test and analysis of variance (ANOVA). DAP and MTAP groups with chitosan showed a significant reduction in the microbial load when compared to calcium hydroxide with chitosan.

Conclusion: DAP and MTAP with chitosan can be effective intracanal medicaments against *E. faecalis* in refractory endodontic cases.

## Introduction

The role of microorganisms has been documented both in the initiation as well as the progression of pulp and periapical pathology. This has been seen in animal models as well as human studies [1]. The ultimate goal in endodontics is to eliminate the bacteria from the root canal system and to prevent the regrowth of any residual organisms [2]. The difficulty in achieving this objective is due to variations in anatomy in the root canal system, making the microorganisms difficult to access by instrumentation and irrigants. Intracanal medicaments help in controlling the increase in microbial load, especially in between appointments [3].

Calcium hydroxide remains the medicament of choice by virtue of its favorable biological properties, especially its high pH and subsequent antimicrobial action [4]. However, several studies have found it to be ineffective against *Enterococcus faecalis*, which seems to be resistant to its antimicrobial effect [5]. As infection of the root canal space is polymicrobial, one single antibiotic cannot be expected to result in effective sterilization of the root canals. The antibiotic combination showing the most promise currently consists of metronidazole, ciprofloxacin, and minocycline [6].

The commonly encountered problem with the use of TAP has been the development of subsequent tooth discoloration, which has been particularly attributed to the minocycline component of the paste [7]. To counter this, the removal of the minocycline component from the paste entirely, production of a double antibiotic paste (DAP), and substitution of the minocycline component with another drug proposed to have similar efficacy, such as clindamycin or cefaclor-modified triple antibiotic paste (MTAP), were the alternatives.

*E. faecalis* is believed to be the most frequently isolated microorganism in re-treatment cases with an incidence of almost 38% [8]. Its inherent capacity to withstand severe environmental changes has been attributed to its high pH tolerance and tubular invasion ability [5].

Chitosan is produced by the partial deacetylation of chitin having multiple applications in the field of medicine and has been used for various purposes like targeted drug delivery, increased absorption of drugs, as a gene delivery agent, and colon targeting [9,10]. The use of chitosan has also been proposed in the field of endodontics as an endodontic irrigant [11,12], for the removal of the smear layer [13], or as a carrier for intracanal medicaments [14].

This study compares the antimicrobial efficacy of DAP, MTAP, and calcium hydroxide with the addition of chitosan nanoparticles against *E. faecalis*. The null hypothesis is that there is no difference in the action of these medicaments on *E. faecalis*.

## Materials and methods

Sixty freshly extracted maxillary and mandibular anterior teeth were collected and stored in distilled water. Single canalled and straight rooted teeth were selected and decoronated using rotary diamond disks keeping a length of 13 mm from coronal to apical end. Teeth were prepared using Gates-Glidden (GG) drill number 3 (Mani, Japan). The debris obtained during the canal preparation was removed by treating the teeth with 17% ethylene diamine tetra acetic acid (RC Help, Prime Dental Products, Mumbai) for 5 min, followed by irrigation with 5% sodium hypochlorite (Dortmund Lab Pvt Limited, Mumbai) for 5 min. The teeth were washed with distilled water for 5 min to remove any traces of the irrigating solutions. All the teeth were completely immersed in individual microcentrifuge tubes (Moxcare Inc., Ambala) containing 1 ml of brain heart infusion (BHI) broth. This entire assembly was then sealed and autoclaved for 20 minutes at 121°C.

Contamination of the teeth

The 24-hour colonies of pure culture of *E. faecalis* were grown on BHI agar and then suspended in 5 ml of BHI broth and incubated for 24 h at 37°C. The 0.5 McFarland standard was used to compare the turbidity changes in the broth, which are an indicator of bacterial growth. This is equivalent to a bacterial suspension of 1.5 × 108 CFU (colony-forming unit)/ml. In a biosafety cabinet, presterilized microcentrifuge tubes having 1 ml of broth were taken, and 50 μl of inocula were added using micropipettes; 5 μl of the broth were subcultured on agar plates from the incubated teeth to ensure the purity of the culture. The duration of the contamination was 21 days.

Preparation of medicaments

Chitosan

It is available in powder form and requires preparation into a solvent form. A 1% solution of acetic acid was prepared by dissolving 1 ml of acetic acid in 99 ml of distilled water. Then, 10 ml of this solution was taken in a beaker, and 0.2 g of chitosan powder (measured on an electronic weighing scale) was added to it. It was stirred vigorously with a glass stirrer for 10-15 minutes and then left overnight to bring about homogenization of the solution, i.e., removal of air bubbles. After 24 hours, a clear homogenous 2% solution of chitosan with a gel-like consistency was obtained.

MTAP and DAP

For MTAP, ciprofloxacin 250 mg, metronidazole 400 mg, and cefaclor tablets were individually ground to a powder form using a mortar and pestle. They were then mixed uniformly in a ratio of 1:1:1 by volume. For DAP, the cefaclor tablet was not included, and the same procedure was followed as stated above. Each paste was mixed with its respective carrier, i.e., saline or chitosan on a glass slab till a suitable consistency was achieved.

Antimicrobial assessment

Teeth were irrigated with 5 ml of sterile saline to remove the incubation broth at 21 days. The teeth were then divided into six groups (n = 10) for receiving the respective medicaments. Five teeth each were chosen for estimation of antimicrobial efficacy (n = 5) on the second and seventh days. One group of 30 teeth had chitosan added to them and another group of 30 teeth had saline added to them: Group D1: DAP + saline, Group D2: DAP + 2% chitosan, Group M1: MTAP + saline, Group M2: MTAP + 2% chitosan, Group C1: CH + saline, and Group C2: CH + 2% chitosan. Temporary restorative material was used for sealing, and the samples were incubated at 37°C.

The assessment was done at the end of two days and seven days with five teeth taken from each group being washed with 5 ml of sterile saline. Dentin debris was collected to a depth of 200 μm using GG drills number 4, and 1 ml of phosphate buffer saline solution was used for storage. The diluted solution was sent for culture with BHI medium. Plates were incubated for 24 hr at 37°C. The number of CFUs between the second day and the seventh day was compared for all the groups.

Statistical analysis

Based on the results of the normality test (Kolmogorov-Smirnov and Shapiro-Wilk tests), it was concluded that the data was not following the normal distribution; hence, nonparametric tests were used. Mann-Whitney U test was used to find the significance of study parameters on a continuous scale between two groups. The Kruskal-Wallis test was used to find the significance of study parameters between three or more groups. Analysis of variance (ANOVA) was used to find the significance of study parameters between the groups (intergroup analysis). Further post hoc analysis was carried out if the values of the ANOVA test were significant. The level of significance was fixed at p = 0.05, and any value less than or equal to 0.05 was considered to be statistically significant.

## Results

On day 2

All antibiotic groups had significantly lower CFU counts than the CH groups, irrespective of the carrier used (chitosan or saline). Among the calcium hydroxide (CH) groups, CH + saline produced a significantly lower CFU count as compared to CH + chitosan. Among the antibiotic groups, DAP produced significantly lower CFU counts when mixed with saline as compared to chitosan. MTAP also produced lower CFU counts when mixed with saline as compared to chitosan, but this difference did not reach statistical significance (p > 0.05) (Table 1).

**Table 1:** Comparison of antimicrobial efficacy in terms of mean (SD) of different medicaments on day 2 using ANOVA test *Statistically significant.

Groups	N (%)	Mean	Std. deviation
Calcium hydroxide + saline	5 (16.6%)	19.40*	3.435
Double antibiotic paste + saline	5 (16.6%)	6.00*	1.414
Modified triple antibiotic paste + saline	5 (16.6%)	4.20	0.447
Calcium hydroxide + chitosan	5 (16.6%)	38.00	4.301
Double antibiotic paste + chitosan	5 (16.6%)	12.40	1.817
Modified triple antibiotic paste + chitosan	5 (16.6%)	4.80	1.924
Total	30	14.13	1.404

On day 7

All groups produced significantly lower counts on day 7 as compared to day 1 (Table 2). There was no statistically significant difference between any of the groups on day 7, with the exception of the CH + chitosan group which still produced significantly higher CFU counts as compared to the others.

**Table 2:** Comparison of the antimicrobial efficacy in terms of mean (SD) of different medicaments on day 7 using the Kruskal-Wallis test

Groups	N (%)	Mean	Std. deviation
Calcium hydroxide + saline	5 (16.6%)	4.40	1.517
Double antibiotic paste + saline	5 (16.6%)	1.20	1.789
Modified triple antibiotic paste + saline	5 (16.6%)	1.00	1.414
Calcium hydroxide + chitosan	5 (16.6%)	16.80*	4.438
Double antibiotic paste + chitosan	5 (16.6%)	0.80	0.837
Modified triple antibiotic paste + chitosan	5 (16.6%)	2.80	2.168
Total	30	4.5	1.260

## Discussion

The presence of pathogenic bacteria is the primary initiating factor for pulpal and periapical infections [15]. Therefore, a decrease in the number of endodontic microorganisms is directly related to the success of root canal treatment. Secondary infection is seen in cases of failed endodontic treatment, where bacteria either persists or re-infects the root canal system after treatment. In canals undergoing re-treatment, canals with persistent infections, or cases of failed endodontic therapy, the two species that have been most commonly isolated include *E. faecalis* and *Candida albicans* [15]. Studies on the prevalence of *E. faecalis* in failed root canals have reported a range between 32% and 70% [16]. *Enterococci *are facultative anaerobes and are extremely resistant to conventional endodontic treatment. They have the capacity to withstand harsh growth conditions, including temperatures ranging from 10˚C to 45˚C, acidic or alkaline environments, and hypo- or hypertonic conditions [15].

Mechanical instrumentation by itself does not result in the complete elimination of bacteria from the root canal system. In vitro studies and clinical evidence have shown that many areas in the canal walls do not get cleaned after mechanical instrumentation [16]. Hence, it seems justified to conclude that the complete elimination of bacteria from the root canal system will not be possible by instrumentation alone. Mechanical instrumentation supplemented with irrigation and intracanal medicaments helps to decrease the number of bacteria/microorganisms in the root canal system.

The antimicrobial activity of calcium hydroxide is due to the release of hydroxyl (OH) ions in an aqueous environment. The OH- are oxidant free radicals that are highly reactive with other biomolecules. Effects on the bacteria include cytoplasmic membrane damage, protein denaturation, and DNA damage [17]. CH is available in the form of water-based or oil-based formulations. An important property imparted by the carrier to the medicament is that of wetting ability or flow. For optimal effect, there should be action of the medicament in eliminating bacteria from the main canal and all ramifications of the canal system. For this, it is important for the medicament to have a lesser contact angle to ensure adequate flow in radicular dentin [3].

The water-based or hygroscopic pastes have superior flow but are associated with higher rates of ionic dissociation and present with some inherent disadvantages. At times, the consistency and mobility of the paste give the operator the impression that the root canal has been filled completely, whereas, in reality, the apical part of the canal is devoid of paste. For this reason, many operators prefer the vehicle for the calcium hydroxide to be oily. The viscous vehicles also allow the paste to remain in the canal for extended periods so that the dressing may be changed less frequently, thereby requiring fewer appointments [18]. On the other hand, oily vehicles are seldom used due to their high viscosity which makes them difficult to remove from the root canal. Their residue on the canal walls may interfere with the adherence of the sealer or obturating material, and thus their use is not recommended [18].

The use of calcium hydroxide is associated with an increased incidence of tooth or root fracture, especially in teeth with open apices that have been treated for overextended time periods for apexification procedures. It has been suggested by researchers that the increased alkalinity after calcium hydroxide placement decreases the bond strength between the dentinal collagen fibrils and the hydroxyapatite [19]. It also exerts a proteolytic effect by increasing the matrix metalloproteinase activity and brings about a conformational change in the structure of the proteoglycan molecules [19]. CH is bactericidal against common endodontic pathogens but less effective against *E. faecalis* or *C. albicans* [4].

"Lesion sterilization and tissue repair (LSTR)" therapy was introduced in 1990 at Niigata University, Japan. This concept uses antibacterial drugs for the disinfection of infectious lesions in the oral cavity in which pulpal and periradicular lesions are included [20]. Ciprofloxacin, a synthetic derivative from the floroquinolone family, is effective against gram-negative and gram-positive bacteria but ineffective against anaerobic ones. Metronidazole is highly effective against anaerobic bacteria, while cefaclor, a β-lactam drug, has a spectrum of action that includes gram-positive aerobes, gram-negative aerobes, and some anaerobic bacteria.

A topical application of antibiotic combinations in the root canal offers several advantages including efficient and predictable disinfection as well as a high concentration of the active agents at the local site. LSTR therapy makes use of small concentrations of antibiotics, and there have been no reported side effects so far. The main side effects of the local use of antibiotics in endodontic infections are hypersensitivity reactions.

Among the vehicles suggested for use with antibiotic pastes, Hoshino et al. [21] recommended propylene glycol and macrogol, while Cruz et al. [22] favored Safranin O dye in propylene glycol. Chitosan can be used in various forms as a carrier as given below.

Gels

Gels are injectable, semisolid formulations having an adequate concentration of drug that can be delivered at a specific site. Its primary advantage lies in its ease of preparation and administration as well as its bioadhesivity, which helps enhance the retention time of the medicament. This form was used in this study.

Microparticulate system

Here, the active drug is encapsulated within micrometer-sized polymer molecules that dissolve slowly, thereby releasing the active agent at the target site. It forms an extremely stable system.

Nanoparticulate system

It has superior dispersion in an aqueous medium, increased stability, controlled rate of release, and better penetration ensuring effective action against a wide spectrum of endodontic pathogens. The disadvantage is that the effectiveness is reduced against specific species such as *E. faecalis* or *C. albicans* [4].

Despite having all of the above-stated advantages, chitosan is a weak base (PKA = 6.2-7) and therefore shows limited solubility at physiological pH of 7.4. Conversion of the glucosamine having -NH2 groups into soluble protonated polycations forming NH3+ ions is possible only in acidic solutions [23]. Chitosan also has a high tendency to swell in aqueous environments, resulting in the fast release of drugs as opposed to the controlled release required in drug delivery applications. This highlights the need for customized chitosan for various applications. Chitosan was used as a carrier in this study to investigate the potential increase it could offer to the antimicrobial effect of calcium hydroxide. Elsaka et al. [24] compared various concentrations (0.5%, 1%, and 2%) of chitosan in combination with calcium hydroxide and found that the 2% concentration had the maximum antibacterial efficacy against *E. faecalis*.

In the present study, the results were obtained by the quantification of the CFUs. This method has a better simulation of the clinical scenario, in which the bacterial strains colonize the radicular dentin surface. Since the medicament/irrigant is allowed to flow into a root canal, it allows for the consideration of other factors that may be important in a clinical setting, i.e., flow properties of the agent, ability to penetrate lateral canals, and handling properties of the agent. It allows for the collection of shavings from different depths into dentin for culturing and thereby allows evaluation of the effect at various depths into the dentin.

In the present study, the effectiveness of CH with chitosan was the least. P/L mixing was carried out to replicate the commonly used method in clinical scenarios with powders of any form (DAP/MTAP or CH). The CH powder showed poor miscibility in this vehicle and produced a grainy, incohesive mix. Decreased flow characteristics of such a formulation could be responsible for its decreased antibacterial effect. In comparison to the CH groups, this study found that antibiotic paste groups produced significantly lower CFU counts of *E. faecalis*. When compared among each other, there was no statistically significant difference between the antibiotic groups. These findings are especially relevant from a clinical perspective as they allow for the removal of the minocycline component of TAP, thereby eliminating the risk of tooth discoloration without adversely affecting the antibacterial properties. The entire workflow according to the Preferred Reporting Items for Lab Studies in Endodontology (PRILE) [25] is given in Table 3.

**Table 3:** PRILE 2021 guidelines PRILE: Preferred Reporting Items for Lab Studies in Endodontology; MTAP: Modified triple antibiotic paste; DAP: Double antibiotic paste; CFU: Colony-forming unit.

Rationale	Addition of chitosan nanoparticles to antibiotic pastes and its role in disinfection of the canal system
Aim	To check the antimicrobial efficacy of chitosan nanoparticles with MTAP, DAP, and calcium hydroxide (CH)
Samples	60 anterior teeth, both maxillary and mandibular, single canalled and straight rooted teeth
Experimental and control groups	Addition of chitosan nanoparticles to MTAP, DAP, CH (n = 30); addition of saline to MTAP, DAP, CH (n = 30); subgroups: D1 – DAP + saline (n = 10); D2 – DAP + chitosan (n = 10); M1 – MTAP + saline (n = 10); M2 – MTAP + chitosan (n = 10); C1 – CH + saline (n = 10); C2 – CH + chitosan (n = 10)
Outcome	Antimicrobial efficacy was checked with *Enterococcus faecalis *strain. Measurement of CFU/ml with an assessment of turbidity
Results	Day 2: MTAP and DAP had lower CFU counts than CH (with both chitosan and saline); DAP and saline were significantly better than DAP + chitosan; Day 7: CH + chitosan had significantly higher CFU counts than other groups
Conclusion	MTAP and DAP mixed with chitosan nanoparticles are useful adjuncts in canal disinfection. The addition of chitosan to MTAP and DAP had a positive antimicrobial effect on *E. faecalis*
Funding details	Nil
Conflict of interest	Nil

## Conclusions

The use of antibiotic pastes is a useful adjunct in the disinfection of the canal system, especially in cases of endodontic re-treatment. The antibiotics are selected in a way that they cover the entire gamut of the microbial spectrum. The addition of chitosan nanoparticles has a positive effect on the effectiveness of these pastes; DAP or MTAP produced a significantly greater reduction in *E. faecalis *counts as compared to CH, irrespective of the carrier used (saline or chitosan). Chitosan when used in the gel form had minimal antibacterial effect on its own when used with the medicaments. The direction for future research should be in the form of finding out an effective formulation of chitosan and antibiotic pastes, which has good handling, better wettability, and improved efficacy.
